# Renal Cell Carcinoma Metastasis to the Breast: A Rare Presentation

**DOI:** 10.1155/2021/6625689

**Published:** 2021-05-07

**Authors:** Heba O. E. Ali, Tamer Ghorab, Iain R. Cameron, Ahmed M. S. M. Marzouk

**Affiliations:** ^1^Altnagelvin Area Hospital, WHSC Trust, UK; ^2^Faculty of Medicine, Cairo University, Egypt

## Abstract

Worldwide breast malignancy is the most common cancer in women; however, metastases to the breast from extramammary malignancies are very rare and only a few sporadic cases are reported in the international literature. In this article, the authors report a case of a 73-year-old woman, who underwent nephrectomy for clear cell renal cell carcinoma and 3 years later presented with a breast metastasis from renal cell carcinoma (clear cell type).

## 1. Introduction

Globally, renal cell carcinoma accounts for 2% of all neoplasms in adults. In 2019, 74,000 new cases of renal carcinoma were diagnosed according to the surveillance, epidemiology, and end results (SEER) statistics report accounting for 4.2%—double universal average—of all cancer diagnoses [1, 2].

About thirty percent of the patients diagnosed with renal cell carcinoma have metastasis at the time of diagnosis [1]. Despite surgical resection, more than 20-50% will progress to metastatic disease [3]. The lung (70%), lymph nodes (55%), bone (42%), liver (41%), adrenal gland (15%), and central nervous system (11%) represent the most common sites of metastasis [4, 5]. Breast metastasis from extramammary primary tumors is extremely rare and ranges from 0.5% to 2% [6, 7]. Melanoma, lymphoma, and leukemia are the most common primary tumors with metastasis to the breast [8]. In the literature, there are limited reports of metastases from renal carcinoma in the breast. In almost half of these cases, metastasis was the first sign [8]. Breast secondaries mimic breast carcinoma in clinical examination, though the diagnosis of extramammary metastasis is vital to avoid unnecessary mastectomy [9].

We present a rare case of primary renal carcinoma metastasis to the breast 3 years after nephrectomy. This is the first case to be reported from Northern Ireland.

## 2. Case Report

**Figure 1:**
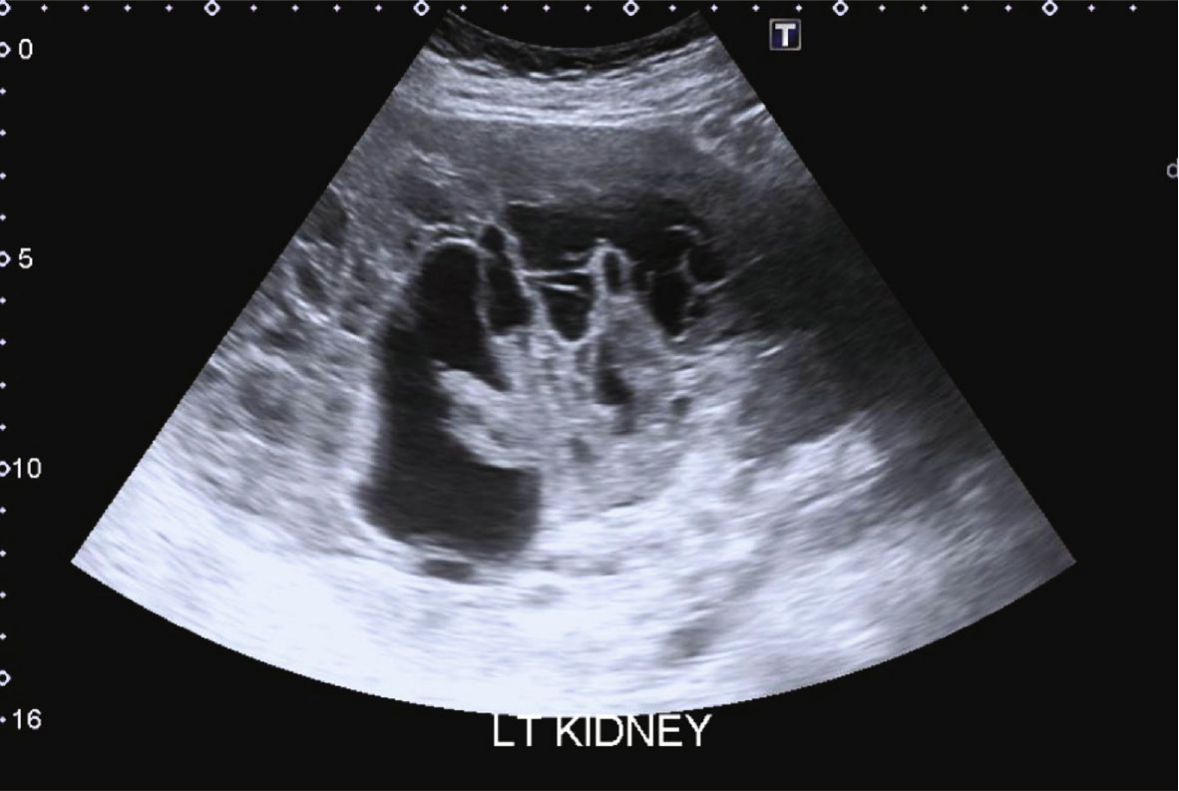
Ultrasound showing malignant looking left renal mass with mixed solid and cystic component.

**Figure 2:**
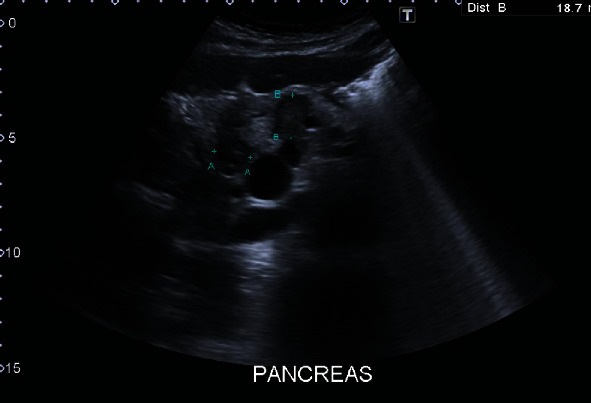
Ultrasound showing hypoechoic masses within the pancreas.

A 73-year-old woman presented with persistent mid and lower abdominal pain. She is known to have IBS, and blood investigations showed iron deficiency anemia. At the time of presentation, the patient had no relevant history of medical comorbidities nor history of smoking; National Early Warning Score (NEWS) was 1 (blood tests outlined in Table 1). She was referred for abdominopelvic ultrasonography which revealed left renal mass around 18 cm with a mixed solid and cystic component; also, hypoechoic foci were noted within the pancreas (Figures 1 and 2).

**Figure 3:**
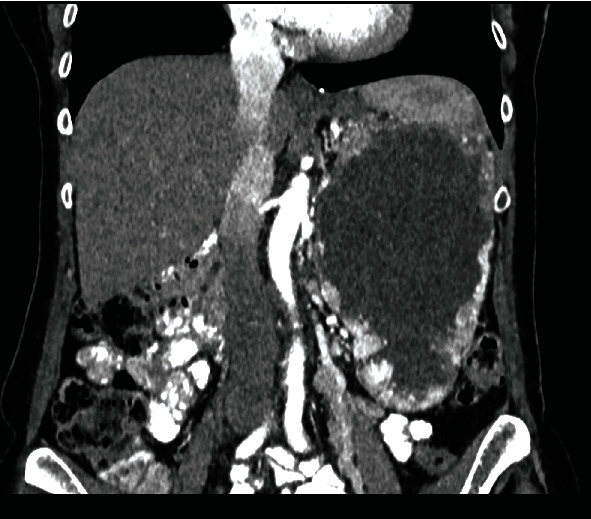
CT showing confirmed a large malignant looking left renal mass with central necrosis.

Subsequent chest CT and CT urogram were done and confirmed a large left renal mass of 18 cm × 10 cm × 12 cm with central necrosis and peripheral hypervascularity (Figure 3). The left renal vein was patent. The adrenal gland looked separable from the mass. Multiple hypervascular foci were noted within the pancreas. Small lung nodules were also noted. Multidisciplinary team (MDT) decision was for cytoreductive nephrectomy and to consider systemic therapy.

A month later, the patient had an open left nephrectomy. Renal cell carcinoma, clear cell type grade II, with no lymphovascular invasion was evident in pathological assessment; surgical margins were clear; and the tumor was classified as grade II pT3a clear cell renal cancer with evident renal vein involvement and no involvement of Gerota's fascia, adrenal gland, perinephric fat, or adjacent organs.

She had evidence of a small volume of metastatic disease in her preoperative imaging. Follow-up every 3 months was scheduled; interval scans in between guided by the clinical examination were done.

**Figure 4:**
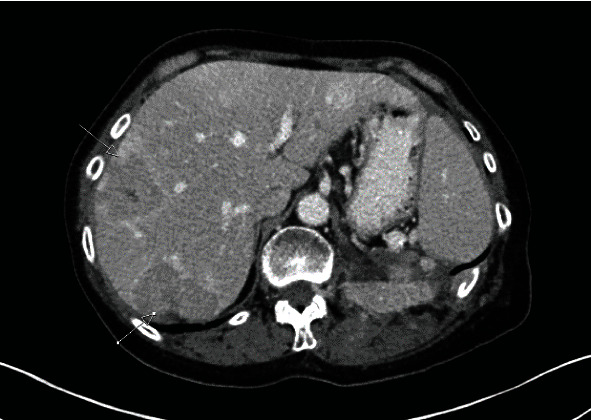
Follow-up CT of the abdomen showing liver deposits.

**Figure 5:**
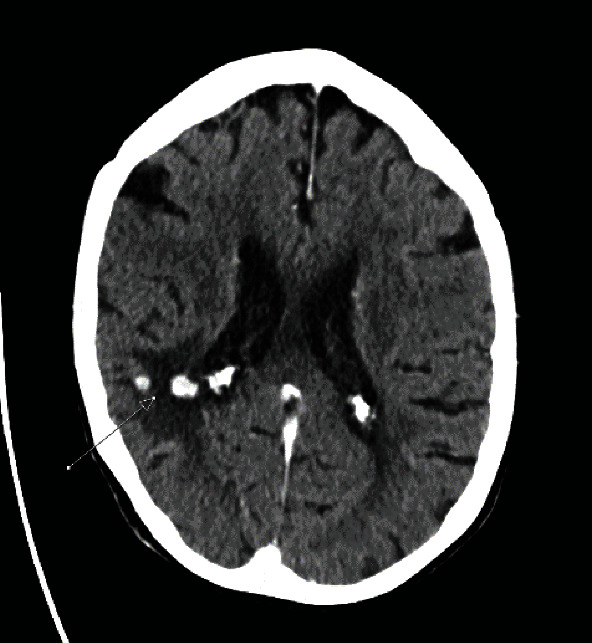
CT of the brain with contrast showing brain metastasis with surrounding edema.

Initially, the patient had shown stable minimal progress of the metastatic disease for which systemic therapy was not indicated at that time. 16-month postnephrectomy, CT follow-up revealed stable chest disease with new hepatic metastasis for which she started sunitinib treatment (Figure 4). Three months later, brain CT showed brain metastasis after she got an episode of confusion (Figure 5). So full head radiotherapy was started. Further progression of the metastatic disease and recurrence of the primary with new deposits in the spine were noted for which radiotherapy was indicated.

At the same time, the patient was referred on red flag breast clinic for assessment of a newly felt lump in her right breast. On examination, the lump was in the upper part of the right breast 1-2 cm, hard, and poorly mobile with no signs of fixation to the covering skin or the underlying muscle. There was fullness in the axillae but no discrete palpable nodes. The patient stated that she had a positive family history of breast cancer.

**Figure 6:**
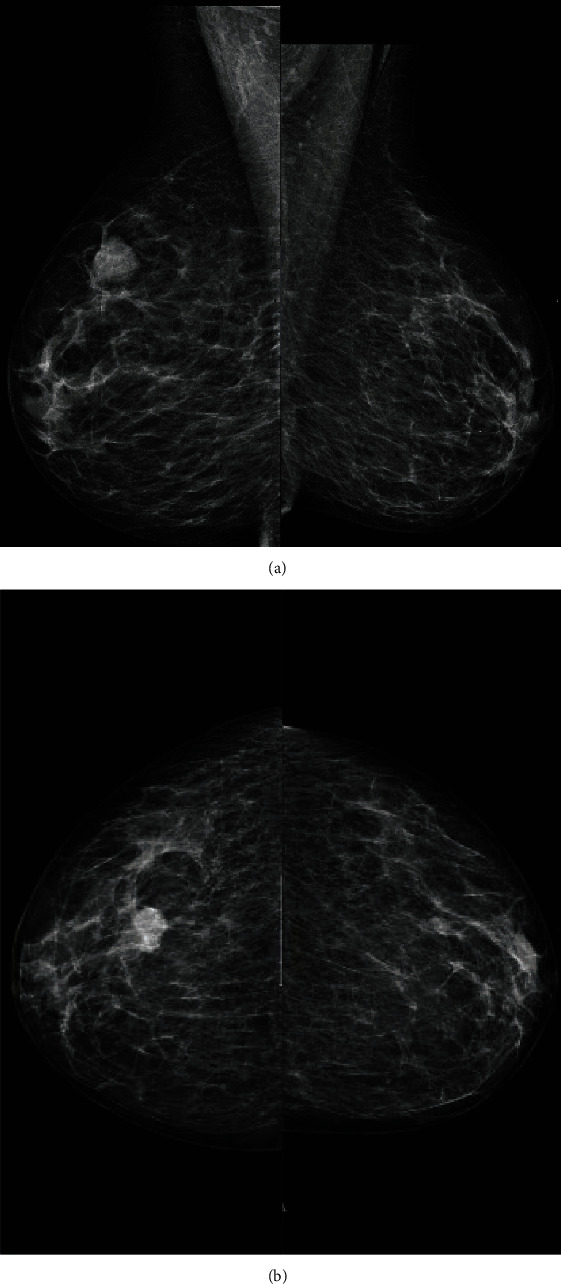
(a) Bilateral mammogram MLO view showing rounded density in the upper part of the right breast. (b) Mammogram in CC view showing a rounded right breast dense lesion.

**Figure 7:**
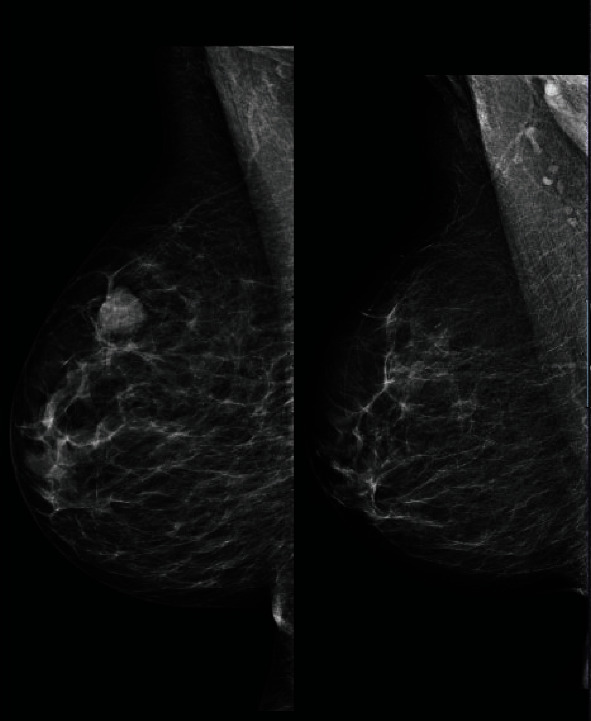
New lesion as compared to previous mammogram.

**Figure 8:**
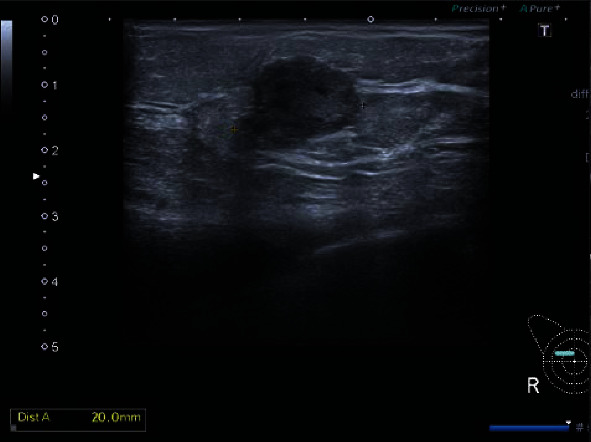
US showing a hypoechoic solid mass lesion in the right breast at 1 o'clock.

Bilateral mammography showed a dense new mass lesion as compared to her previous mammogram done 2 years earlier (Figures 6 and 7). The mass was seen in the upper half of the right breast without microcalcifications. Since it was a new finding, M4 score was given. Breast ultrasound (US) confirmed a rather well-defined hypoechoic solid mass lesion in the right breast at 1 o'clock measuring 20 mm × 13 mm with tiny cystic changes. Axilla was normal. It was reported as U5 in ultrasound. US-guided core biopsy was done in the same clinic (Figure 8).

Even though the patient had widespread RCC metastatic disease, it was important to clarify if this lesion represented a primary breast tumor which should indicate endocrine therapy (if ER-positive) or a secondary from the RCC which should be managed conservatively unless getting symptomatic.

**Figure 9:**
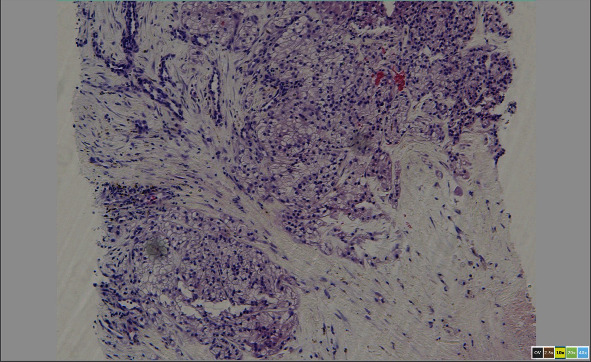
The standard H&E-stained section showing cells with small nuclei and abundant clear cytoplasm typical of clear cell RCC.

**Figure 10:**
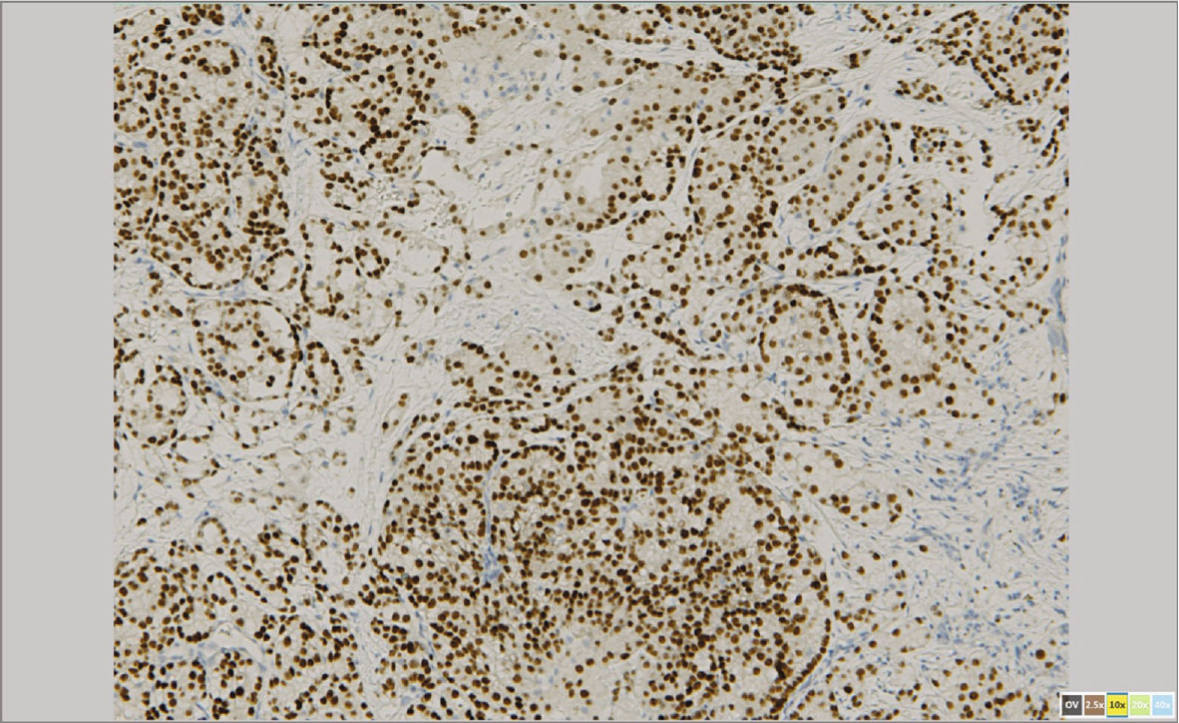
Image shows positive immunohistochemical cell nucleus staining with PAX8.

**Figure 11:**
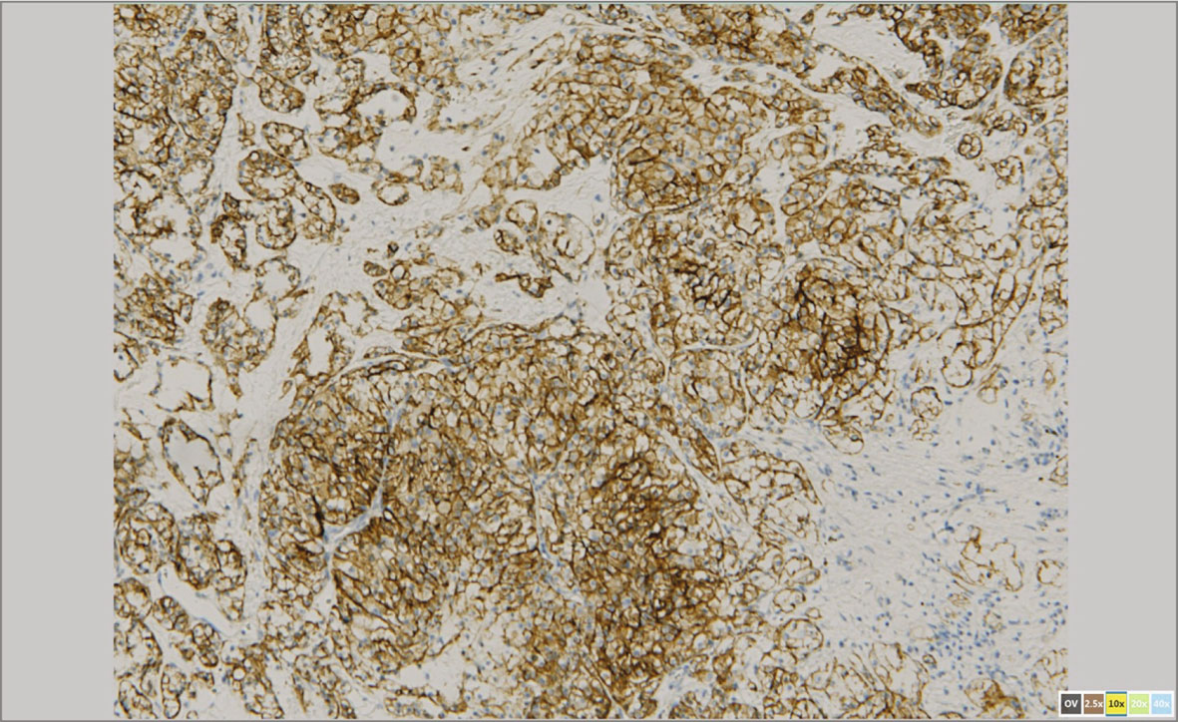
Image shows positive cell membrane staining with carbonic anhydrase.

Pathology revealed breast tissue infiltrated by tumor with a nested pattern composed of clear cells. A panel of immunohistochemical markers reveals positive staining with the renal markers PAX 8 and carbonic anhydrase (Figures 9–11). CK7 is negative as are the breast markers GATA 3 and mammaglobin. Given the clinical history, the features were those of metastatic renal cell carcinoma.

## 3. Discussion

The breast is considered a rare site for metastatic lesions with reported incidence up to 2% and 6.6% in autopsy series of all malignant neoplasms [6, 9, 10]. It is primarily a female condition, close to that associated with primary breast cancer [11]. The most frequent metastases to the breast are from malignant melanoma, lymphoma, lung cancer, and, in men, prostatic cancer [9, 12]. In the literature, there is scanty evidence of the prognosis in these patients who has metastatic disease in the breast [13]. However, proper differentiation between primary breast neoplasms and metastatic breast disease is essential to establish an appropriate management plan.

Approximately 25%–30% of patients with RCC will present with metastatic disease at the time of diagnosis. Despite that the lung, bone, regional lymph nodes, liver, and brain are the most common sites of metastases [14], renal cell carcinoma is able to metastasize in unusual organs such as the thyroid, pancreas, spleen, or vagina either individually or in the form of multimetastatic disease [15–18]. Micrometastases formation and subsequent colonization require complex adaptations by tumor cells to various host components [19]. “Seed and Soil” theory represents the ability of RCC metastasis to unusual organs [15]. Metastases to the breast from RCC are rare. The first reported RCC metastasis to the breast was in the early 4th decade of the twentieth century, and only 25 cases have been reported in the literature [14].

Smoking is a major risk factor for cancer process including growth and metastasis. Both particulate and gas carcinogens act on the cell cycle, oncogene, or antioncogene loosening in addition to cell death process alteration with resultant abnormal cell growth and tumor invasiveness [20]. A large retrospective study found smoking had relative risk of 1.6-fold of RCC [1]. In this case, the patient had no history of smoking or known risk factors explaining the behaviour of disease progression.

History of extramammary tumors should be considered during evaluation of suspicious breast lesion; combined approach of clinical history, examination, and investigations is important to achieve proper diagnosis, which in turn influences proper management plan and prognosis [21]. Differentiation of primary breast cancer from secondary metastasis was vital in this patient as it was necessary to guide the management. Although primary breast malignancy is a common presentation in such age, however, the history of current metastatic disease elevated the index of suspicion.

Pathological assessment represents an integral part of the multidisciplinary approach in such situations. Their abilities to relevant immunohistochemical markers are of great importance to differentiate primary breast neoplasms from metastasis [21]. Immunohistochemical markers in this patient revealed positive staining with the renal markers PAX 8 and carbonic anhydrase and negative CK7, GATA 3, and mammaglobin.

As part of the triple assessment, the mammographic appearance of metastatic breast cancer is usually well defined with a lack of microcalcifications and a history of malignancy should increase the suspicion of metastasis [13, 14, 22]. In fact, the optimum treatment is controversial [13, 23]. Mastectomy and lymph node dissection are unnecessary because they are not a curative treatment. Palliative radiotherapy and target therapy are good local and systemic control with tolerable toxicity [13, 22, 23].

Surgical management (mastectomy and lymph node dissection) in such condition does not offer curative option; palliative options as radiotherapy and target therapy offer good and systemic and local control with endurable toxicity [9, 13, 22, 23].

In this case, surgery was only indicated in case of being symptomatic. This patient had disseminated metastasis (breast, pulmonary, liver, spine, and brain) besides the local recurrence. There were no effective therapeutic options for such disseminated disease. Based on a multidisciplinary approach, palliative care was followed.

There is minimal information in the literature regarding the prognosis of patients with metastatic disease to the breast, and further studies are warranted [9].

In conclusion, breast metastasis from RCC is very rare. Primary and secondary breast cancer may be clinical and radiological. Triple assessment (clinical, radiological, and histological evaluation) is mandatory for confirmation of the diagnosis. An individualized and multidisciplinary approach of such patients should be followed.

## Figures and Tables

**Table 1:** Blood test parameters at the time of presentation.

Haemoglobin estimation	98^∗^	775-745 (g/L)	Sodium	137	136-145 (mmol/L)
Hct	0.313^∗^	0.37-0.47	Potassium	4.3	3.5-5.3 (mmol/L)
Red blood cell (RBC) count	3.64^∗^	3.8-5.8 (e12/L)			
			Chloride	104	95-108 (mmol/L)
Mean corpuscular volume (MCV)	86.0	76-96 (fL)			
Mean corpusc. haemoglobin (MCH)	26.9^∗^	27-32 (pg)			
			CO_2_	25	22-29 (mmol/L)
Mean corpusc. Hb. conc. (MCHC)	313	300-350 (g/L)			
			Urea	4.2	2.5-7.8 (mmol/L)
Red blood cell distribution width	16.3^∗^	9.0-15.5			
			Creatinine	78	45-84 (*μ*mol/L)
Platelet count	280	150-440 (e9/L)			
			Calcium	2.10	2.10-2.60 (mmol/L)
Pct	—	(%)			
			Magnesium	0.84	0.7-1.0 (mmol/L)
Mean platelet volume	9.2	(fL)			
			Albumin	31^∗^	35-50 (g/L)
Platelet distribution width	9.8				
			Phosphate	1.24	0.8-1.5 (mmol/L)
Total white cell count	9.0	4.0-11.0 (e9/L)			
			ALT	23	<33 (U/L)
Percentage lymphocytes	16.2	(%)			
			AST	17	<32 (U/L)
Percentage monocytes	12.7	(%)			
			GGT	90^∗^	6-42 (U/L)
Percentage granulocytes	—	(%)			
			ALP	15	30-130 (U/L)
LY^#^	1.46	1.0-4.8 (e9/L)			
			T. bilirubin	3	<21 (*μ*mol/L)
Monocyte count	1.15^∗^	0-0.8 (e9/L)			
			LDH (IFCC)	151	§
GR^#^	—	(e9/L)			
			Adjusted calcium	2.32	2.20-2.60 (mmol/L)
Percentage neutrophils	69.6	(%)			
			eGFR	>60	(mL/min/1.73 m^2^)
Percentage eosinophils	0.7	(%)			
			Serum iron level	9^∗^	13-30 (*μ*mol/L)
Percentage basophils	0.8	(%)			
			Serum transferrin	1.5^∗^	1.8-3.8 (g/L)
Neutrophil count	6.29	1.8-7.7 (e9/L)			
			Serum ferritin	402^∗^	13-300 (*μ*g/L)
Eosinophil count	0.06	0-0.60 (e9/L)			
			Transferrin saturation index	24	§
Basophil count	0.07	0-0.2 (e9/L)			

Asterisks indicate abnormal findings.
